# The mechanism of arsenic trioxide and microwave ablation in the treatment of oral squamous cell carcinoma based on high throughput sequencing

**DOI:** 10.1049/syb2.12113

**Published:** 2024-12-23

**Authors:** Xuesong Zhang, Yakun Liu, Shengteng He, Liangjia Bi, Bing Liu

**Affiliations:** ^1^ Department of Stomatology The 962nd Hospital of the PLA Joint Logistic Support Force Harbin China; ^2^ Department of Stomatology The Fourth Affiliated Hospital Harbin Medical University Harbin China; ^3^ Department of Stomatology Heilongjiang Nursing College Harbin China; ^4^ Department of Stomatology Hainan Provincial Third People's Hospital Sanya China; ^5^ Department of Oral and Maxillofacial Surgery The First Affiliated Hospital Harbin Medical University Harbin China; ^6^ School of Stomatology Harbin Medical University Harbin China

**Keywords:** bioinformatics, RNA, tumours

## Abstract

Oral squamous cell carcinoma (OSCC) is a common head and neck malignant tumour with high incidence and poor prognosis. Arsenic trioxide (ATO) has therapeutic effects on solid tumours. Microwave ablation (MWA) has unique advantages in the treatment of solid tumours. However, the therapeutic mechanism of ATO and MWA, as well as their combined effect on OSCC were largely unelucidated. Cal‐27 cell‐bearing nude mice were treated with ATO and/or MWA, respectively. RNA sequencing was used to obtain gene expression profiles in tumour tissues of mice treated by ATO or MWA. RNA sequencing results were verified by real‐time polymerase chain reaction (PCR). The lncRNA‐miRNA‐mRNA co‐expression network was constructed based on the competitive endogenous RNA (ceRNA) theory. Gene ontology and Kyoto Encyclopedia of Genes and Genomes analyses were performed using differentially expressed genes. The combined effect of ATO and MWA on OSCC was evaluated. Finally, CCK‐8 assay, EdU assay and transwell migration assay were performed to detect the effect of HSPA6 on the proliferation and migration of OSCC cells. The reduced volume of tumour tissues was observed in both ATO‐ and MWA‐treated groups. 37.8% decreased in the ATO group and 35.0% in the MWA group. A total of 207 and 539 differentially expressed mRNAs and lncRNAs were identified in the ATO group. And a total of 200 and 522 differentially expressed mRNAs and lncRNAs in the MWA group were identified. The expression levels of 8 genes were verified by real‐time PCR. The differentially expressed genes were closely related to “chemical carcinogenesis”, “herpes simplex infection”, “porphyrin and chlorophyll metabolism”, and “MAPK signalling pathway”. The lncRNA‐miRNA‐mRNA co‐expression networks were constructed. The combined treatment with ATO and MWA showed a better inhibitive effect on OSCC than either of them. The synergistic effect of ATO and MWA was related to the upregulation of HSPA6. The downregulation of HSPA6 could promote the proliferation and migration of OSCC cells. This study detected the long non‐coding RNA and mRNA expression profiles related to the treatment of OSCC and constructed corresponding ceRNA networks. Arsenic trioxide and MWA have a synergistic effect on OSCC, which was related to the upregulation of HSPA6.

## Graphical abstract

This study detected the lncRNA and mRNA expression profiles related to the treatment of OSCC and constructed corresponding ceRNA networks. ATO and MWA have a synergistic effect on OSCC, which was related to the upregulation of HSPA6.

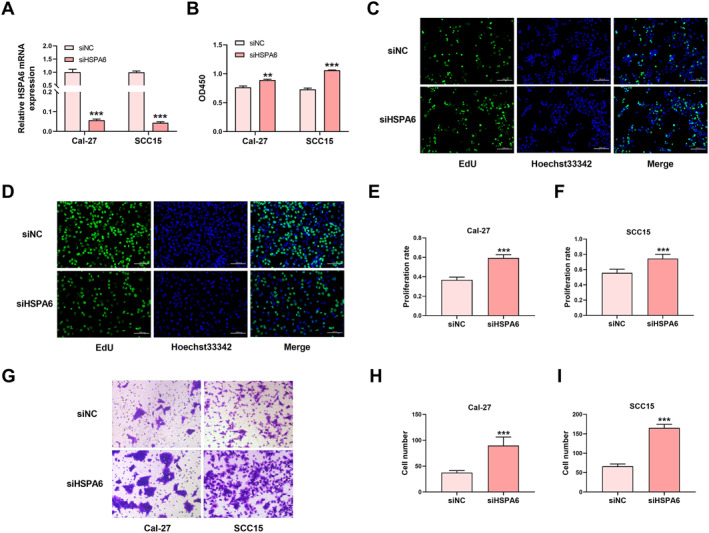

## 1 INTRODUCTION

Oral squamous cell carcinoma (OSCC) is a common head and neck malignant tumour with high incidence [1]. Despite the progression of surgery treatment, radiotherapy, and chemotherapy technology, most late‐stage OSCC patients have a poor prognosis. The 5‐year survival rate of OSCC is only about 50%, and the survival rate has not improved significantly in recent decades [2]. Therefore, finding new treatment targets and exploring new treatment strategies for OSCC are of great significance.

Arsenic trioxide (ATO) is a first‐line treatment for acute promyelocytic leukemia (APL) [3]. It also has therapeutic effects on many solid tumours including breast cancer, prostate cancer, liver cancer etc [4, 5, 6]. In recent years, studies have shown that ATO has a significant inhibitory effect on human oral cancer cells [7]. Arsenic trioxide can inhibit the proliferation and colony formation and promote the apoptosis of oral cancer cells, which was related to the inhibition of VEGF pathways [8]. In addition, ATO could also enhance the anti‐cancer effect of cisplatin [9]. However, the therapeutic mechanism of ATO on oral cancer is still not fully understood.

Microwave ablation (MWA) is a new technology for the treatment of tumours through local heating. Its principle is to put a microwave antenna inside the tumour and heat the tumour tissues through high‐frequency microwave, thus inducing necrotic death of tumour cells. The pain during the treatment is slight, and general anaesthesia is not required. In addition, the postoperative recovery is rapid, which can significantly reduce the occurrence of complications. In recent years, MWA technology has achieved great success in the treatment of solid tumours. At present, the technology has been applied to the treatment of liver cancer [10], kidney cancer [11], lung cancer [12], pancreatic cancer [13], osteosarcoma [14], breast cancer [15] etc. Since the application of MWA may have a risk of incomplete ablation, some researchers have combined anti‐tumour drugs with MWA, which showed a synergistic effect [16]. Therefore, MWA may also enhance the therapeutic effect of ATO on OSCC.

Non‐coding RNA is closely related to the occurrence and development of a variety of diseases [17, 18]. Long non‐coding RNA (lncRNA) is a kind of nucleotides longer than 200 bp, which is without protein coding property. Studies have shown that the abnormal expression of lncRNA is associated with the pathogenesis and progression of a variety of tumours [19, 20]. In recent years, lncRNA was shown to be involved in the mechanism of competitive endogenous RNA (ceRNA). That is to say, lncRNA can act as a sponge of miRNA, thus regulating the expression of coding genes [21]. Many studies have shown that lncRNAs are involved in the proliferation, apoptosis, metastasis, and invasion of OSCC through a variety of molecular mechanisms [22, 23].

The objective of this study was to investigate the therapeutic mechanisms of ATO and MWA in OSCC by analysing gene expression profiles, constructing lncRNA‐miRNA‐mRNA co‐expression networks, evaluating the combined effect of ATO and MWA, and assessing the role of HSPA6 in OSCC cell proliferation and migration in order to improve the understanding of OSCC treatment and identify potential therapeutic targets.

## 2 MATERIALS AND METHODS

### 2.1 Establishment of xenograft model of nude mice

BALB/c nude mice were purchased from Beijing Vital River Laboratory Animal Technology Co., Ltd. (Beijing, China). Cal‐27 cells were purchased from Shanghai Institute of Cells (Chinese Academy of Sciences), which are epithelial cells isolated from a patient with tongue squamous cell carcinoma. Cal‐27 cell suspension (0.2 mL) at the density of 1 × 10^7^/ml was injected into the right armpit of the nude mice subcutaneously [24]. We recorded the volume of tumour every day. One week after injection, nodules appeared under the skin of the nude mice. The treatment was carried out when the length and diameter of the tumour reached 9–10 mm. The nude mice in the ATO group were given ATO 5 mg/kg every day with an injection volume of 0.2 mL, and the control group was given an equal volume of normal saline for 2 weeks. The power of the MWA group was set to 5 W and the duration time was 2 min. The combined treatment group was treated with both MWA and ATO.

### 2.2 mRNA and Long non‐coding RNA sequencing

Total RNA was extracted from tumour tissue samples. Each group has three repetitions. The constructed gene library was sequenced using Illumina NovaSeq 6000. The transcript was reconstructed by StringTie. The coding ability of new transcripts was predicted using two software CPC2 [25] and CNCI [26], and the intersection of these transcripts without coding potential was taken as a reliable new lncRNA prediction result. The expression of genes in each sample was calculated by comparing the expression of gene in HISAT2. The mRNA and lncRNA expression profiles were obtained based on gene expression in each sample. Differentially expressed genes were analysed by DESeq2 software [27]. The mRNAs and lncRNAs with *P* < 0.05 and |FC| > 1.5 were collected.

### 2.3 Real‐time PCR analysis

TRIzol reagent was used to extract total RNA from tissue samples and cells according to the manufacturer's protocol. Briefly, the samples were homogenised in the TRIzol reagent, and then chloroform was added to separate the aqueous phase containing RNA from the organic phase. The aqueous phase was subsequently transferred to a fresh tube, and RNA was precipitated with isopropanol. After washing with ethanol, the RNA was air‐dried and dissolved in RNase‐free water. The purity and concentration of the extracted RNA were evaluated by spectrophotometry, ensuring high‐quality RNA for downstream applications. The first‐strand of cDNA was synthesised by using a reverse transcriptase kit (Roche, Mannheim, Germany). Reverse transcription was performed using Hiscript II Q RT SuperMix for qPCR (Vazyme Biotech Co., Ltd., Nanjing, China). The real‐time polymerase chain reaction (PCR) reaction mixture was prepared according to the manufacturer's instructions, containing cDNA template, forward and reverse primers, SYBR green master mix (for qPCR detection), and RNase‐free water. The reaction mixture was then loaded into the ABI StepOne Plus system (Applied Biosystems, CA, USA), and the thermal cycling conditions were optimised for each primer pair to achieve the best amplification efficiency. The primer sequences were as follows: HSPA6, 5′‐ATCCAGAGGAACGCCACTA‐3′ (forward), 5′‐TGCCACTGAGTTCAAAACG‐3′ (reverse); TBC1D31, 5′‐ATCGATACGGAACCAAGCA‐3′ (forward), 5′‐GAATGCATCCTCCTCAGGAA‐3′ (reverse); SNHG1, 5′‐TCTGGAATCTACCTGCCCTTT‐3′ (forward), 5′‐TCTGGGCCTGGATCATGTAA‐3′ (reverse); GARS1‐DT, 5′‐GGCAGTGCCTGTTGACATAG‐3′ (forward), 5′‐CTACCTGCTACTGCGAGACTT‐3′ (reverse); TMEM147‐AS1, 5′‐CGAAACCAGTCTCTCCCTCTTA‐3′ (forward), 5′‐TTGATGGTGAGTTGAGGATGAG‐3’ (reverse); JPX, 5′‐GGCCCATTGGAATTGTTAC‐3′ (forward), 5′‐CTCTTCTCGGTGCCTAATCT‐3′ (reverse); GAPDH, 5′‐GGGAAACTGTGGCGTGAT‐3′ (forward), 5′‐GAGTGGGTGTCGCTGTTGA‐3′ (reverse). GAPDH was used as the internal control. Target gene expression was analysed using the 2 ^‐∆∆CT^ method.

### 2.4 Enrichment analysis

Gene ontology (GO) [28] and Kyoto Encyclopedia of Genes and Genomes (KEGG) [29] databases were used for enrichment analysis. A hypergeometric test was performed to compare the whole genomes and screen GO entries or KEGG pathways which significantly enriched in candidate genes.

### 2.5 Competitive endogenous RNA network construction

We analysed the expression data of lncRNAs and mRNAs to calculate the Pearson correlation coefficient for each pair. Significant interactions (|PCC| > 0.5) were retained for network construction. Based on the significant lncRNA‐mRNA interactions, we generated a ceRNA network. The network was visualised using Cytoscape [30], where nodes represent lncRNAs and mRNAs, and edges indicate their correlation.

### 2.6 Cell culture and transfection

Cal‐27 and SCC15 cells were cultured using RPMI‐1640 (Procell) with 10% FBS (Excell Bio). The siRNA sequences targeting HSPA6 were purchased from Tsingke Biotechnology Co., Ltd. The siRNA was transfected into cells using Lipofectamine 2000 (invitrogen,Cat.No. 11668019). The sequence of selected siRNA for HSPA6 was as follows: sense CGUGUUGCGGAUCAUCAAU (dT)(dT); and antisense AUUGAUGAUCCGCAACACG (dT)(dT).

### 2.7 CCK‐8 assay

The cells were seeded in the 96‐well plate and transfected with siNC or siHSPA6. After treatment, cell viability was detected by cell counting kit‐8 (Beyotime Biotechnology).

### 2.8 EdU staining

The proliferation ability of cells was detected by EdU staining (Guangzhou RiboBio Co., LTD). After treatment, the cells were fixed with 4% paraformaldehyde and stained with EdU and Hoechst 33342 regents. The procedure was according to the manufacturer's instructions.

### 2.9 Transwell migration assay

The cells were seeded in the upper chamber of the transwell plate (BD, REF353097). After 48 h of treatment, the migrated cells were fixed with 4% paraformaldehyde and stained with crystal violet. The cell images were obtained and the number of migrated cells was calculated.

### 2.10 Statistical analysis

Statistical comparisons between two groups were performed by using Student's *t*‐test. Data from more than two groups were analysed by one‐way ANOVA. *P* < 0.05 was considered statistically significant.

## 3 RESULTS

### 3.1 Effects of Arsenic trioxide and Microwave ablation on OSCC‐bearing nude mice

**FIGURE 1:**
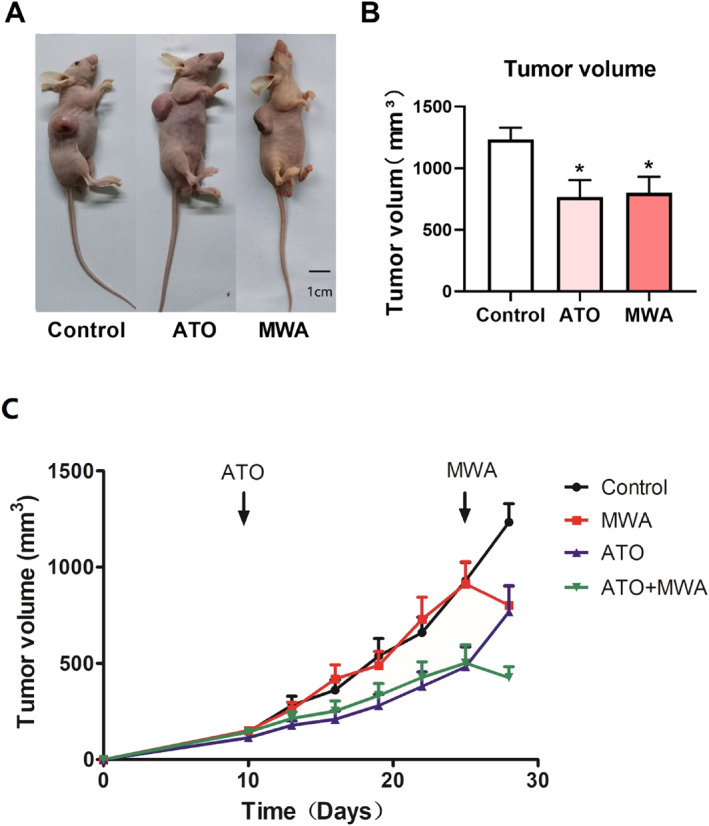
The effect of ATO and MWA on OSCC‐bearing nude mice. (a) Representative images of nude mice in control, ATO, and MWA groups. (b) The tumour volume of nude mice in control, ATO, and MWA groups. (c) Change in the tumour volume of Cal‐27 xenograft tumours in nude mouse during the treatment period. *P < 0.05; versus control; *n* = 6. ATO, arsenic trioxide; MWA, microwave ablation; OSCC, oral squamous cell carcinoma.

Cal‐27 cells were used to establish an OSCC‐bearing nude mice model. The mice were treated by ATO or MWA. At the end of the experiment, the volume of tumour was measured. The volume of tumours in nude mice from the control group was larger than that in the ATO and MWA groups (Figure 1a and b). Change in the tumour volume of Cal‐27 xenograft tumours in nude mouse during the treatment period was shown in Figure 1c. These results indicated that ATO and MWA treatments both have inhibitory effects on OSCC.

### 3.2 Expression profile of mRNA and Long noncoding RNA related to Arsenic trioxide treatment in OSCC‐bearing nude mice

**FIGURE 2:**
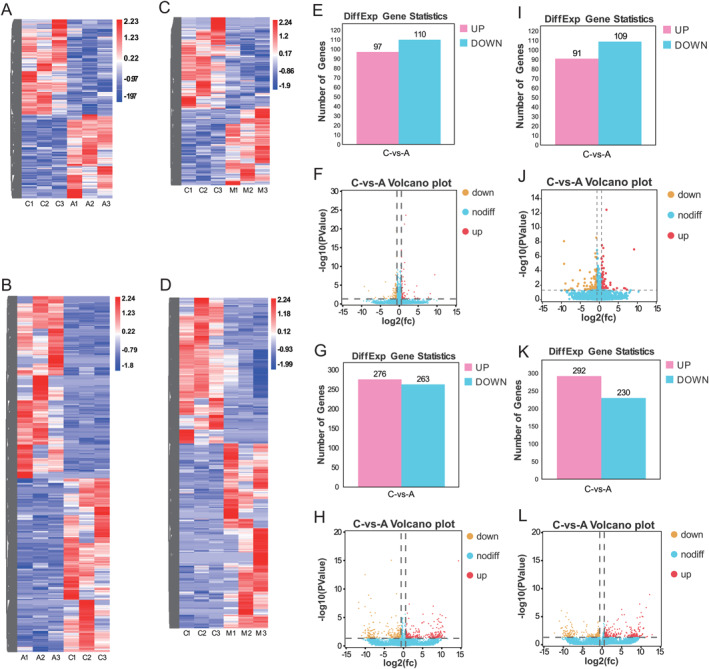
Differentially expressed mRNAs and lncRNAs in ATO and MWA groups. (a) and (b) The heatmap of mRNA (a) and lncRNA (b) in control and ATO groups. (c) and (d) The heatmap of mRNA (c) and lncRNA (d) in control and MWA groups. (e) and (f) The number (e) and volcano plots (f) of differentially expressed mRNAs in ATO groups. (g) and (h) The number (g) and volcano plots (h) of differentially expressed lncRNAs in ATO groups. (i) and (j) The number (i) and volcano plots (j) of differentially expressed mRNAs in MWA groups. (K and L) The number (g) and volcano plots (h) of differentially expressed lncRNAs in MWA groups. (a) Represents the ATO group (c) Represents the control group M: Represents the MWA group. ATO, arsenic trioxide; lncRNA, long non‐coding RNA; MWA, microwave ablation.

RNA sequencing was used to obtain the gene expression profile in tumour tissues of mice treated by ATO. The differentially expressed genes between the two groups were screened by *P* < 0.05 and |FC| > 1.5. The gene expression heatmaps and volcano plots were shown in Figure 2a, b, f, and h. A total of 207 (97 upregulated and 110 downregulated) and 539 (276 upregulated and 263 downregulated) differentially expressed mRNAs and lncRNAs were identified between control and ATO groups (Figure 2e and g). The detailed gene expression profiles were provided in the supplementary data.

### 3.3 Expression profile of mRNA and Long noncoding RNA related to Microwave ablation treatment in OSCC‐bearing nude mice

RNA sequencing was used to obtain the gene expression profile in tumour tissues of mice treated by MWA. The differentially expressed genes between the two groups were screened by *P* < 0.05 and |FC| > 1.5. The gene expression heatmaps and volcano plots were shown in Figure 2 (c, d, j, and l). A total of 200 (91 upregulated and 109 downregulated) and 522 (292 upregulated and 230 downregulated) differentially expressed mRNAs and lncRNAs were identified between control and ATO groups (Figure 2i and k). The detailed gene expression profiles were provided in the supplementary data.

### 3.4 The expression of mRNAs and lncRNAs verified by real‐time PCR

**FIGURE 3:**
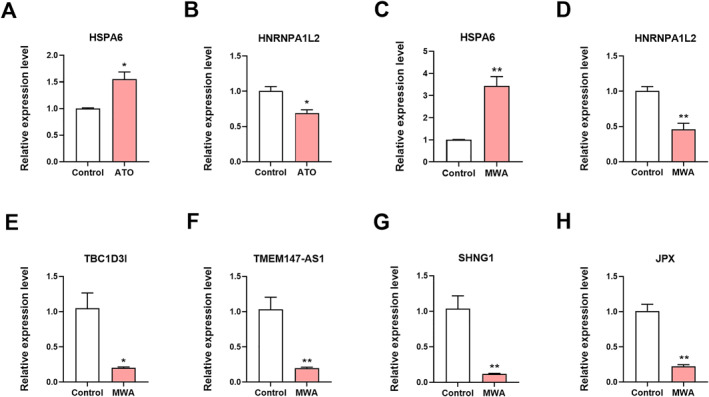
Real‐time PCR verification of the differentially expressed mRNAs and lncRNAs in ATO and MWA groups. (a)–(b) The mRNA expression levels of HSPA6 (a), HNRNPA1L2 (b) in control and ATO groups. (c)–(h) The mRNA expression levels of HSPA6 (c), HNRNPA1L2 (d), TBC1D3I (e), TMEM47‐AS1 (f), SHNG1 (g), and JPX (h) in control and MWA groups. *P < 0.05; **p < 0.01; versus control; *n* = 3. ATO, arsenic trioxide; MWA, microwave ablation; PCR, polymerase chain reaction.

Real‐time PCR was used to verify the RNA sequencing results. The expression levels of HSPA6, HNRNPA1L2, TBC1D3I, TMEM147‐AS1, SHNG1, and JPX were detected (Figure 3a–h). The real‐time PCR results were consistent with the RNA sequencing results.

### 3.5 Enrichment analysis of differentially expressed mRNAs in Arsenic trioxide group

**FIGURE 4:**
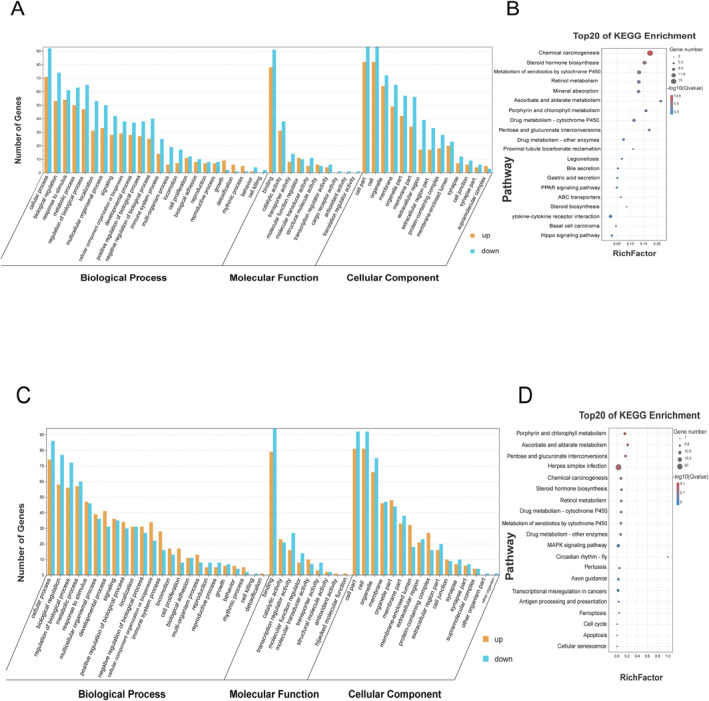
Enrichment analysis of differentially expressed genes in ATO and MWA groups. (a) GO enrichment analysis of differentially expressed genes in control and ATO groups. (b) KEGG enrichment analysis of differentially expressed genes in control and ATO groups. (c) GO enrichment analysis of differentially expressed genes in control and MWA groups. (d) KEGG enrichment analysis of differentially expressed genes in control and MWA groups. ATO, arsenic trioxide; GO, gene ontology; KEGG, Kyoto Encyclopedia of Genes and Genomes; MWA, microwave ablation.

A total of 207 differentially expressed mRNAs in the ATO group were enriched by GO and KEGG analyses. It was found that these genes were closely related to biological processes such as “Cellular process”, “Biological regulation”, and “Response to stimulus” and to signal pathways such as “Chemical carcinogenesis”, “Steroid hormone biosynthesis”, “Retinol metabolism”, and “PPAR” (Figure 4a and b).

### 3.6 Enrichment analysis of differentially expressed mRNAs in Microwave ablation group

A total of 200 differentially expressed mRNAs in the MWA group were enriched by GO and KEGG analyses. The results showed that these genes were closely related to biological processes such as “Cellular process”, “Biological regulation”, and “Regulation of biological process”, and were closely related to signal pathways such as “Chemical carcinogenesis”, “Herpes simplex infection”, “Porphyrin and chlorophyll metabolism”, and “MAPK signalling pathway” (Figure 4c and d).

### 3.7 Construction of Arsenic trioxide and MWA‐related competitive endogenous RNA networks

**FIGURE 5:**
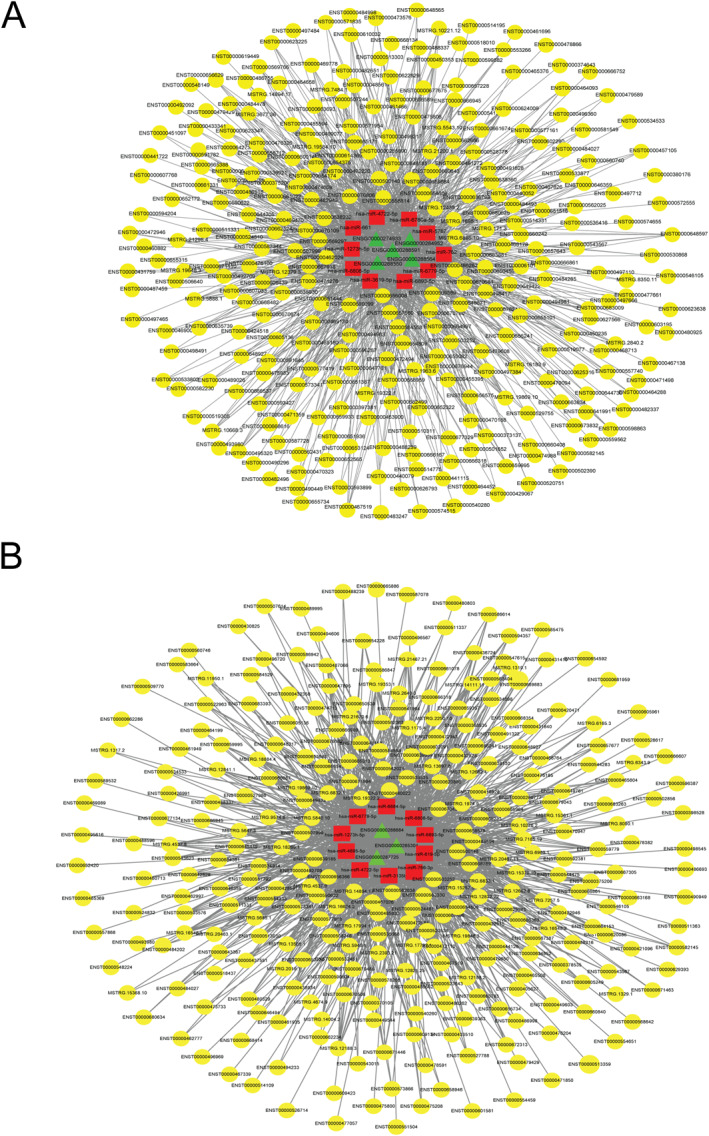
Construction of ATO and MWA‐related ceRNA networks. (a) The ATO‐related ceRNA network. (b) The MWA‐related ceRNA network. Green triangle, red square, and yellow circle represent mRNA, miRNA, and lncRNA, respectively. ATO, arsenic trioxide; ceRNA, competitive endogenous RNA; lncRNA, long non‐coding RNA; MWA, microwave ablation.

Based on the ceRNA theory, we established the lncRNA‐miRNA‐mRNA ceRNA networks for ATO and MWA, respectively. The ATO‐related ceRNA network has 310 nodes (including 295 lncRNAs, 10 miRNAs, and 5 mRNAs) and 1317 edges (Figure 5a), while the MWA‐related ceRNA network has 311 nodes (including 298 lncRNAs, 10 miRNAs, and 3 mRNAs) and 1341 edges (Figure 5b).

### 3.8 The combined effect of Arsenic trioxide and Microwave ablation on OSCC‐bearing nude mice

**FIGURE 6:**
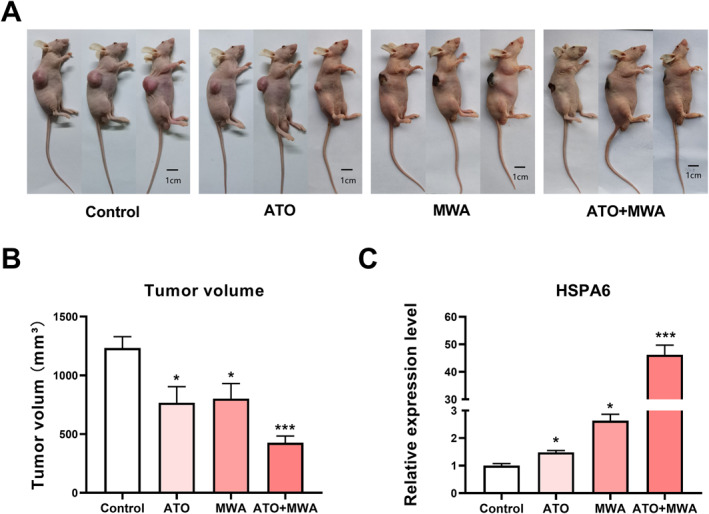
The combined effect of ATO and MWA on OSCC. (a) Representative images of nude mice in control, ATO, MWA, and ATO + MWA groups. (b) The tumour volume of nude mice in control, ATO, MWA, and ATO + MWA groups. (c) The mRNA expression of HSPA6 in control, ATO, MWA, and ATO + MWA groups. *P < 0.05, ***P < 0.001 versus control; *n* = 3–6. ATO, arsenic trioxide; MWA, microwave ablation; OSCC, oral squamous cell carcinoma.

Subsequently, the combined effect of ATO and MWA on OSCC‐bearing nude mice was evaluated. The combined treatment with ATO and MWA showed a high inhibitive effect on OSCC than either of them (Figure 6a and b). Real‐time PCR was used to verify the expression of HSPA6 mRNA in the tumour tissues. The expression of HSPA6 mRNA in the combination group was higher than that in the ATO and MWA groups. These results suggest that ATO and MWA have a synergistic effect on OSCC, which was related to the upregulation of HSPA6 (Figure 6c).

### 3.9 The effect of HSPA6 inhibition on the proliferation and migration of Oral squamous cell carcinoma cells

**FIGURE 7:**
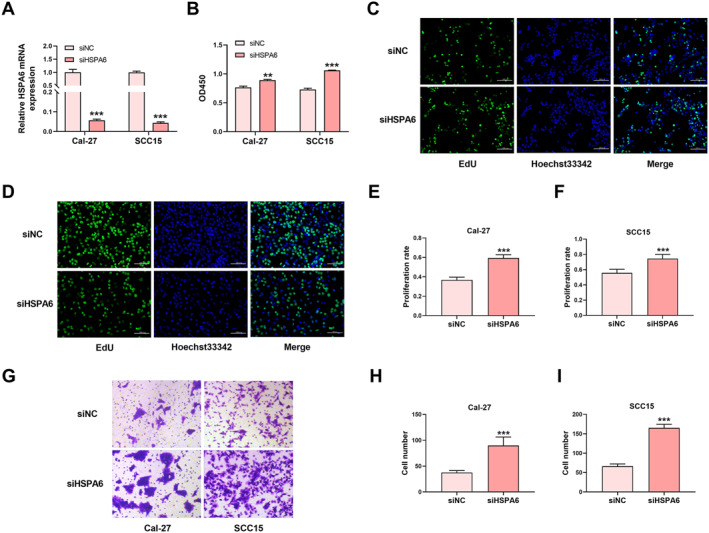
The effect of HSPA6 inhibition on the proliferation and migration of OSCC cells. (a) Transfection of siHSPA6 could inhibit the expression of HSPA6 in Cal‐27 and SCC15 cells. (b) The effect of HSPA6 inhibition on the viability of Cal‐27 and SCC15 cells. (c) and (d) Representative EdU staining images of Cal‐27 (c) and SCC15 (d) cells. (e) and (f) Statistical results of EdU staining of Cal‐27 (e) and SCC15 (f) cells. (g) Representative transwell migration assay images of Cal‐27 and SCC15 cells. (h) and (i) Statistical results of transwell migration assay images of Cal‐27 cells (h) and SCC15 cells (i). **P < 0.01, ***P < 0.001 versus control; *n* = 3–8. OSCC, oral squamous cell carcinoma.

Finally, we designed siRNAs targeting HSPA6. The most effective siRNA sequence was selected for the following experiments (Figure 7a). CCK‐8 assay and EdU staining showed that the inhibition of HSPA6 promoted the proliferation of Cal‐27 and SCC15 cells (Figure 7b–f). The transwell migration assay showed that the inhibition of HSPA6 promoted the migration of Cal‐27 and SCC15 cells (Figure 7g–i).

## 4 DISCUSSION

Oral squamous cell carcinoma has been recognised as a global public health problem [31]. However, surgery destroys the appearance of patient and seriously affects the life quality of patients [32]. Many patients diagnosed with advanced malignant tumours often lost the opportunity of surgery. The toxic and side effects of drugs caused by radiotherapy and chemotherapy also limit the application in tumour therapy. Therefore, finding a new treatment strategy for OSCC is vitally important.

Arsenic trioxide, as a specific drug in the treatment of APL, has therapeutic effects on many solid tumours including breast cancer [33], renal cell carcinoma [34], prostate cancer [35], and liver cancer [36]. Microwave ablation is a new minimally invasive technique for the treatment of tumours by local heating. Ultrasound‐guided MWA palliative treatment of advanced head and neck malignant tumours is a safe and effective palliative treatment method, which helps to reduce the symptom of patients, improve quality of life, and prolong life time. Microwave ablation may also have a therapeutic effect on OSCC.

In our study, Cal‐27 cell‐bearing nude mice were treated with ATO and/or MWA, respectively. RNA sequencing was used to obtain a gene expression profile in tumour tissues of mice treated by ATO or MWA. The differentially expressed genes were screened by *P* < 0.05 and |FC| > 1.5. A total of 207 and 539 differentially expressed mRNAs and lncRNAs were identified in the ATO group. And a total of 200 and 522 differentially expressed mRNAs and lncRNAs in the MWA group were identified. The expression levels of some genes were verified by real‐time PCR. The results of real‐time PCR were consistent with that of RNA sequencing.

Then, enrichment analysis was performed using differentially expressed genes. It was found that the differentially expressed mRNAs in the ATO group were closely related to pathways including “Chemical carcinogenesis”, “Steroid hormone biosynthesis”, “Retinol metabolism”, and “PPAR”. While, mRNAs in the MWA group were closely related to pathways including “Chemical carcinogenesis”, “Herpes simplex infection”, “Porphyrin and chlorophyll metabolism”, and “MAPK signalling pathway”, some of these pathways play important roles in OSCC. For example, steroid hormones are highly expressed in OSCC cells, which may promote the carcinogenesis of OSCC [37]. The activation of ERK/MAPK pathway has an inhibitory effect on OSCC [38, 39].

Based on the ceRNA theory, we established the lncRNA‐miRNA‐mRNA ceRNA networks for ATO and MWA, respectively. Through the constructed ceRNA networks, we found that the expression of TBC1D31 mRNA was decreased in ATO and MWA groups, the expression of lncRNA SNHG1 was decreased in these two groups, and the expression of lncRNA JPX was decreased in these two groups. The study found that key node miR‐762 may play an important role in ATO and MWA groups. MiR‐762 was involved in pathways including lncRNA SNHG1‐miR‐762‐TBC1D31 and lncRNA JPX‐miR‐762‐TBC1D31, which may be related to the treatment of OSCC.

The cancer promoting effect of lncRNA SNHG1 and JPX in cancer has been validated. Zhao et al. [40] found that SNHG1 overexpression promoted cell proliferation, migration and invasion in OSCC cells. The deletion of SNHG1 inhibited cell migration and invasion accompanied with decreased MMP2 and MMP9 protein expression. Huang et al. [41] found that SNHG1 was significantly up‐regulated in prostate cancer tissues and cells. Knockout of SNHG1 significantly inhibited the proliferation, migration, and invasion, which promoted apoptosis of prostate cancer cells. Yao et al. [42] found that lncRNA JPX is highly expressed in OSCC cells and could promote tumours progression through the miR‐944/CDH2 axis. Silencing lncRNA JPX inhibited the proliferation, migration, and invasion of OSCC cells. Chen et al. [43] found that JPX was significantly up‐regulated in cervical cancer tissues and cell lines, which could promote the proliferation, migration and invasion of Hela cells.

Subsequently, we measured the combined effect of ATO and MWA on OSCC‐bearing nude mice. The combined treatment with ATO and MWA showed a stronger inhibitive effect on OSCC than either of them. Through gene expression profile analysis, we found that the mRNA expression level of the HSPA6 gene significantly increased in the ATO and MWA groups compared with that in control. The expression level of HSPA6 mRNA in the combination group was higher than that in the ATO group and MWA group. It has been reported that HSPA6 has an inhibitory effect on the growth, migration, and invasion of three negative breast cancer cells. The high expression of HSPA6 is positively correlated with the overall survival of breast cancer patients, suggesting that HSPA6 has a tumour suppressor effect [44]. These results suggest that ATO and MWA have a synergistic effect on OSCC, which was related to the upregulation of HSPA6. Finally, we explored the effect of HSPA6 on OSCC cells. The inhibition of HSPA6 could promote the proliferation and migration of OSCC cells. A small sample size is the limitation of our work. Larger sample sizes and human trials are needed to validate our findings. Future research should address this limitation to enhance the robustness and applicability of our results.

Our gene sequencing study provides novel insights into ATO and MWA treatments for OSCC. By elucidating RNA interactions and mechanisms, we offer a new treatment paradigm. This gene‐based approach, suitable for advanced OSCC patients, may alleviate financial burdens and enhance quality of life. Our findings also pave the way for targeted, personalised treatments and encourage further research into RNA biology to uncover new therapeutic targets.

## 5 CONCLUSION

In conclusion, our study obtained ATO and MWA treatment‐related gene expression profiles and constructed the corresponding regulatory network for OSCC. Arsenic trioxide and MWA have a synergistic effect on OSCC, which was related to the upregulation of HSPA6. Revealing the potential molecular mechanism of the interaction between coding RNA and non‐coding RNA will promote our new understanding of the mechanism of ATO and MWA in the treatment of OSCC and put forward a new treatment strategy. For patients with advanced OSCC who are not suitable for surgical treatment, this relatively cheap treatment strategy has the potential to reduce the economic burden of patients and improve their quality of life.

## AUTHOR CONTRIBUTIONS

Bing Liu and Liangjia Bi participated in the experimental design and critical revision of the paper; Xuesong Zhang performed the in vivo experiments and data analysis and drafted the paper; Yakun Liu provided the investigation and research on experimental methods; Shengteng He participated in software analysis and validation and all authors have given their final approval for the submission of this paper.

## CONFLICT OF INTEREST STATEMENT

The authors declare no competing financial interests.

## Supporting information

The differentially expressed mRNAs between control and arsenic trioxide groups.

The differentially expressed lncRNAs between control and arsenic trioxide groups.

The differentially expressed mRNAs between control and microwave ablation groups.

The differentially expressed lncRNAs between control and microwave ablation groups.

## Data Availability

The data that support the findings of this study are available on request from the corresponding author.
